# Ultrasound features for prediction of long-term outcomes of women with primary breast cancer <20 mm

**DOI:** 10.3389/fonc.2023.1103397

**Published:** 2023-03-16

**Authors:** Sihui Shao, Minghua Yao, Chunxiao Li, Xin Li, Jianfeng Wang, Jing Chen, Yi Zheng, Rong Wu

**Affiliations:** ^1^ Department of Ultrasound, Shanghai General Hospital, Shanghai Jiao Tong University School of Medicine, Shanghai, China; ^2^ Department of General Surgery, Shanghai General Hospital, Shanghai Jiao Tong University School of Medicine, Shanghai, China

**Keywords:** breast cancer, ultrasonography, mammary, early cancer, disease-free survival, cancer specific survival

## Abstract

**Background:**

Some women die despite the favorable prognosis of small breast cancers. Breast ultrasound features may reflect pathological and biological characteristics of a breast tumor. This study aimed to explore whether ultrasound features could identify small breast cancers with poor outcomes.

**Methods:**

This retrospective study examined confirmed breast cancers with a size of <20 mm diagnosed in our hospital between 02/2008 and 08/2019. Clinicopathological and ultrasound features were compared between alive and deceased breast cancer patients. Survival was analyzed using the Kaplan-Meier curves. Multivariable Cox proportional hazards models were used to examine the factors associated with breast cancer-specific survival (BCSS) and disease-free survival (DFS).

**Results:**

Among the 790 patients, the median follow-up was 3.5 years. The deceased group showed higher frequencies of spiculated (36.7% vs. 11.2%, P<0.001), anti-parallel orientation (43.3% vs. 15.4%, P<0.001), and spiculated morphology combined with anti-parallel orientation (30.0% vs. 2.4%, P<0.001). Among 27 patients with spiculated morphology and anti-parallel orientation, nine cancer-specific deaths and 11 recurrences occurred, for a 5-year BCSS of 77.8% and DFS of 66.7%, while 21 breast-cancer deaths and 41 recurrences occurred among the remaining patients with higher 5-year BCSS (97.8%, P<0.001) and DFS (95.4%, P<0.001). Spiculated and anti-parallel orientation (HR=7.45, 95%CI: 3.26-17.00; HR=6.42, 95%CI: 3.19-12.93), age ≥55 years (HR=5.94, 95%CI: 2.24-15.72; HR=1.98, 95%CI: 1.11-3.54), and lymph nodes metastasis (HR=3.99, 95%CI: 1.89-8.43; HR=2.99, 95%CI: 1.71-5.23) were independently associated with poor BCSS and DFS.

**Conclusions:**

Spiculated and anti-parallel orientation at ultrasound are associated with poor BCSS and DFS in patients with primary breast cancer <20 mm.

## Introduction

With the wide implementation of breast cancer imaging screening, an increasing number of small-sized breast cancers are being detected (1–3). Women with small breast cancer generally have favorable long-term outcomes (3–5). Nevertheless, breast cancer is a heterogeneous disease with complicated pathology and biological behavior, and some women eventually die of breast cancer or have early recurrence despite having a small tumor (6, 7). Indeed, tumor size by itself does not tell much about the biological behavior because a specific tumor size can result from an indolent disease that has been growing for some time or from a very aggressive disease that has been developing for a few weeks (5, 6, 8–10). In addition, even though tumor size is a well-recognized prognostic factor for invasive breast cancer, large ductal carcinoma *in situ* (DCIS) carries the risk of harboring microinvasive foci that can lead to poor outcomes (11).

Despite their value in predicting the long-term outcomes, traditional prognostic factors (such as pathological grade and lymph node status) and the more powerful molecular and genetic examinations might not be sufficient for the prognosis of small breast cancer (12–16). Some women with small breast cancer still die from breast cancer (6, 7). Clinicopathological factors like pathological tumor grade and lymph node status are commonly used for breast cancer prognosis (17, 18) but are not as effective in small breast cancers as in larger ones (12–16). Thus, other reliable prognostic factors to identify the more aggressive subset among small breast cancers as early as possible are needed to ensure appropriate management.

Breast ultrasound features may reflect the underlying pathological manifestations and biological behavior of a breast tumor, which would be reliable imaging indicators for breast cancer outcomes (15, 19). Still, most studies focused on assessing the associations between ultrasound features and breast cancer risk categories like molecular type and histological grade (16–18). In contrast, studies examining the direct associations between ultrasound features and breast cancer mortality are insufficient, especially for small breast cancers <20 mm.

Therefore, this study aimed to explore whether ultrasound features could identify small breast cancers with poor outcomes.

## Materials and methods

### Study design and patients

This retrospective study included consecutive patients (including patients who came for the routine examination and patients with symptomatic complaints) with pathologically confirmed breast cancer with a size of <20 mm (histologic size) newly diagnosed in our hospital between February 2008 and August 2019. Only patients with a single lesion were included. Each patient underwent a preoperative whole breast ultrasound examination and received surgical therapy with negative margin.

The exclusion criteria were 1) with a history of breast cancer, 2) suspected or proven metastatic foci, 3) received any type of cancer treatment before surgery, or 4) underwent biopsy without surgery. The minimum follow-up was 12 months.

This retrospective study was approved by the institutional ethics committee of Shanghai General Hospital. Written informed consent was waived by the institutional review board.

### Ultrasound examination

All ultrasound examinations were performed in the routine clinical setting using an iU22 ultrasound system (Philips, Best, The Netherlands) with a 5-12-MHz linear transducer and an APlio 500 ultrasound system (Toshiba Corp., Tokyo, Japan) with a 10-MHz linear transducer. All ultrasound examinations were performed by radiologists with >3 years of experience in breast ultrasound screening.

### Image analysis

For the present study, two radiologists with >3 years of experience in breast ultrasound imaging diagnosis reviewed the stored diagnostic images. The two radiologists were blind to other imaging examinations and the pathological results. The imaging features were analyzed using the BI-RADS lexicon, including shape (oval, round, or irregular), margin (circumscribed, indistinct, microlobulated, angular, or spiculated), orientation (≥1 or <1), presence of calcification or not, posterior features (no change, enhancement, focal acoustic shadow, combined pattern), echo pattern (hyperechoic, hypoechoic or mixed-echoic), and the grade of blood flow (absent, internal vascularity or vessels in the rim). If disagreement occurred, both readers re-assessed the ultrasound features classification and reached an agreement.

### Pathology

All patients underwent surgery in the routine clinical management setting for such lesions as per the inclusion criteria. The results of the routine pathological examinations were defined as the final diagnosis. Tumor grade and lymph node status were assessed. Immunohistochemistry was performed to determine the expression of the estrogen receptor (ER), progesterone receptor (PR), human epidermal receptor 2 (HER2), and Ki67. The cut-off for positive ER and PR was 10% and 14% for positive Ki67. HER2 was defined as positive when 3+ or confirmed as positive using fluorescence *in situ* hybridization (20, 21).

### Data collection and definitions

Patients’ characteristics, pathological tumor characteristics, the primary surgical treatment (mastectomy or quadrantectomy and axillary procedures), and adjuvant therapy (chemotherapy or radiotherapy) were retrospectively collected from the patient charts.

Breast cancer-specific survival (BCSS) was defined as the time from the first diagnosis to breast cancer-specific death. Disease-free survival (DFS) was considered the interval between the first diagnosis and the first evidence of recurrence, metastasis, or new diagnosis of breast carcinoma. For this study, the deaths were carefully reviewed and ruled by the investigators. Patients were divided into the deceased and alive groups based on whether they died of breast cancer or not.

### Statistical analysis

Statistical analysis was performed using SPSS 19.0 (IBM Corp., Armonk, NY, USA). Two-sided P-values <0.05 were considered statistically significant. The ultrasound features of the malignant breast lesions and the clinicopathological characteristics between women with and without breast cancer death were compared using the chi-square test or Fisher’s exact test, as appropriate. Continuous variables were analyzed using Student’s t-test. The survival analyses were carried out using the Kaplan-Meier curves and the log-rank test. Multivariable Cox proportional hazards models were used to confirm the association of ultrasound features and outcomes after adjustment for other existing prognostic factors, including age, pathological grade, lymphovascular invasion, molecular biomarker, and adjuvant therapy. Covariables factors were further analyzed in lesions of 1-10 mm. Hazard ratios (HR) and 95% confidence intervals (CI) were used to describe the predictive factors.

## Results

### Characteristics of the patients

During the study period, 821 women were found with a breast mass <20 mm by histological measurement. Women with a history of breast cancer (n=21), with metastatic foci (n=2), who received treatment before surgery (n=5), or underwent biopsy without surgery (n=3) were excluded. Ultimately, 790 women with breast cancer <20 mm were included. The mean age at diagnosis was 56.3 ± 12.3 years, and the most common pathological type was invasive ductal carcinoma with an early stage, grade I and II, and negative lymph nodes status. The detailed clinicopathological characteristics are listed in **Table 1**.

**Table 1:** The association of histologic findings with survival outcomes.

Characteristics	Total (n=790)	Dead (n=30)	Alive (n=760)	P
Age (years), mean ± SD	56.3 ± 12.3	68.1 ± 13.3	55.8 ± 12.1	<0.001
Pathological type, n (%)				0.634
Intraductal carcinoma in situ	97 (12.3)	2 (6.7)	95 (12.5)	
Invasive ductal carcinoma	594 (75.2)	24 (80.0)	570 (75.0)	
Mucinous breast carcinoma	29 (3.7)	2 (6.7)	27 (3.6)	
Intraductal papillary carcinoma	30 (3.8)	0	30 (3.9)	
Invasive lobular carcinoma	25 (3.1)	1 (3.3)	24 (3.2)	
Others	15 (1.9)	1 (3.3)	14 (1.8)	
Grade, n (%)				0.514
Carcinoma in situ	75 (9.5)	1 (3.3)	74 (9.7)	
I	156 (19.7)	6 (20.0)	150 (19.7)	
II	363 (45.9)	13 (43.3)	350 (46.1)	
III	196 (24.8)	10 (33.3)	186 (24.5)	
Lymph nodes metastasis, n (%)				<0.001
Negative	586 (74.2)	13 (43.3)	573 (75.4)	
Positive	204 (25.8)	17 (56.7)	187 (24.6)	
ER status, n (%)				0.981
Positive	570 (72.2)	21 (70.0)	549 (72.2)	
Negative	215 (27.2)	8 (26.7)	207 (27.2)	
Unknown	5 (0.6)	1 (3.3)	4 (0.5)	
PR status, n (%)				0.068
Positive	452 (57.2)	12 (40.0)	440 (57.9)	
Negative	330 (41.8)	17 (56.7)	313 (41.2)	
Unknown	8 (1.0)	1 (3.3)	7 (0.9)	
HER2 status, n (%)				0.828
Positive	146 (18.5)	6 (20.0)	140 (18.4)	
Negative	617 (78.1)	23 (76.7)	594 (78.2)	
Unknown	27 (3.4)	1 (3.3)	26 (3.4)	
Ki67, n (%)				
Positive	455 (57.6)	16 (53.3)	439 (57.8)	0.720
Negative	265 (33.5)	8 (26.7)	257 (33.8)	
Unknown	70 (8.9)	6 (20.0)	64 (8.4)	
Surgery type, n (%)				0.610
Mastectomy	457 (57.8)	16 (53.3)	441 (58.0)	
Quadrantectomy	333 (42.2)	14 (46.7)	319 (42.0)	
Surgery type of axillary, n (%)				0.484
Axillary dissection	571 (72.3)	20 (66.7)	551 (72.5)	
Axillary biopsy	219 (27.7)	10 (33.3)	209 (27.5)	
Adjuvant therapy, n (%)				0.086
Yes				
Chemotherapy	293 (37.1)	9 (30.0)	284 (37.4)	
Radiotherapy	117 (14.8)	3 (10.0)	114 (15.0)	
Chemotherapy and radiotherapy	52 (6.6)	1 (3.3)	51 (6.7)	
No	328 (41.5)	17 (56.7)	311 (40.9)	

### Survival

The median follow-up for the 790 patients was 3.5 (interquartile range, 2.1-5.5) years, with a 96.2% BCSS and 93.4% DFS. Thirty patients died from breast cancer and four from other causes. When comparing the traditional risk factors between the two groups, age (P<0.001) and lymph nodes metastasis (P<0.001) were associated with breast cancer death. No significant differences were observed regarding the pathological type, histopathological grade, surgery type, adjuvant therapy, ER, PR, HER2, and Ki67, though negative PR was with a borderline P-value (P=0.068) (**Table 1**). There were nine breast cancer deaths, and 11 recurrences occurred among patients with spiculated morphology and anti-parallel orientation, while 21 breast cancer deaths and 41 recurrences events among the remaining.

### Association of ultrasound features with survival outcomes

**Figure 1:**
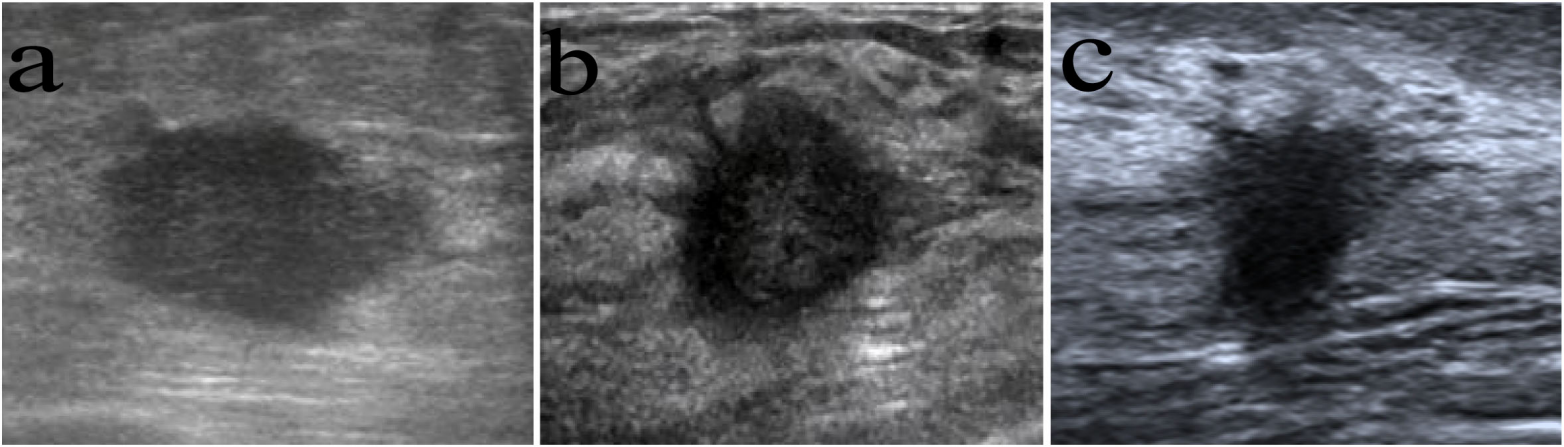
Images in women with different long-term outcomes. **(A)**, **(A)** A 54-year-old woman with breast cancer showing a parallel orientation, without spiculated morphology, and with no recurrence after 82 months of follow-up. **(B)** A 51-year-old woman with breast cancer with spiculated morphology and anti-parallel orientation. She died of distant metastasis 33 months from the first diagnosis. **(C)** A 50-year-old woman with breast cancer with spiculated morphology and anti-parallel orientation. She died of distant metastasis 21 months from the first diagnosis.

**Figure 2:**
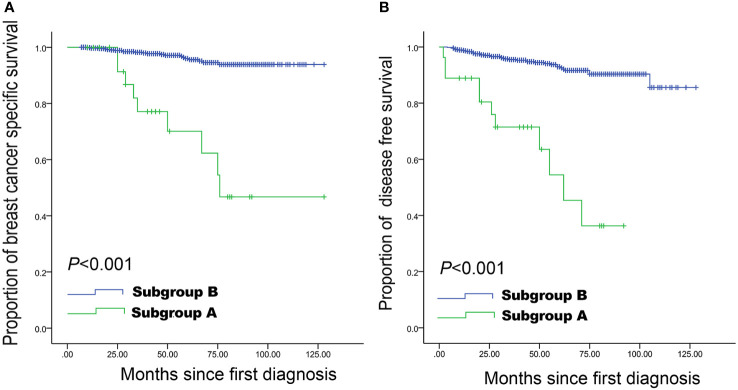
Breast cancer-specific survival and disease-free survival curves in women with small breast cancer. Spiculated morphology and anti-parallel orientation were strongly associated with a lower breast cancer-specific survival **(A)** and disease-free survival **(B)** compared with the remaining women with lesions of 1-20 mm. Subgroup A: Lesions with both spiculated morphology and anti-parallel orientation; Subgroup B: all other patients.

As shown in **Table 2**, the shape, calcification, vascularity, posterior features, and echo patterns were not significantly different between the two groups (all P>0.05), while there were significant difference in the distribution of margin (P=0.006) and orientation (P<0.001) between the two groups. Compared with the survival group, the deceased group showed higher frequencies of spiculated (36.7% vs. 11.2%, P<0.001), anti-parallel orientation (43.3% vs. 15.4%, P<0.001), and spiculated morphology combined with anti-parallel orientation (30.0% vs. 2.4%, P<0.001) (**Table 2** and **Figure 1**). The patients with spiculated morphology and anti-parallel orientation had a lower 5-year BCSS (77.8% vs. 97.8%, P<0.001) and DFS (66.7% vs. 95.4%, P<0.001) (**Figure 2**) than the patients without those two features.

**Table 2:** Association of ultrasound features with survival outcomes.

	Dead (n=30)	Alive (n=760)	P
Shape			0.376
Regular			
Oval	0	38 (5.0)	
Round	3 (10.0)	47 (6.2)	
Irregular	27 (90.0)	675 (88.8)	
Margin			0.006
Circumscribed	6 (20.0)	159 (20.9)	0.903
Indistinct	5 (16.7)	235 (30.9)	0.096
Microlobulated	2 (6.7)	95 (12.5)	0.568
Angular	6 (20.0)	186 (24.5)	0.575
Spiculated	11 (36.7)	85 (11.2)	<0.001
Orientation			<0.001
parallel	17 (56.7)	643 (84.6)	
not parallel	13 (43.3)	117 (15.4)	
Spiculated and anti-parallel orientation	9 (30.0)	18 (2.4)	<0.001
Calcification			0.632
Presence	11 (36.7)	312 (41.1)	
Absence	19 (63.3)	448 (58.9)	
Vascularity			0.456
Absent	16 (53.3)	414 (54.5)	
Internal vascularity	14 (46.7)	302 (39.7)	
Vessels in rim	0	44 (5.8)	
Posterior features			0.229
No change	21 (70.0)	488 (64.2)	
Enhancement	3 (10.0)	105 (13.8)	
Focal acoustic shadow	3 (10.0)	138 (18.2)	
Combined pattern	3 (10.0)	29 (3.8)	
Echo pattern			0.123
Hyperechoic	0	7 (0.9)	
Hypoechoic	27 (90.0)	731 (96.2)	
Heterogenous	3 (10.0)	22 (2.9)	

### Multivariable analysis

**Figure 3:**
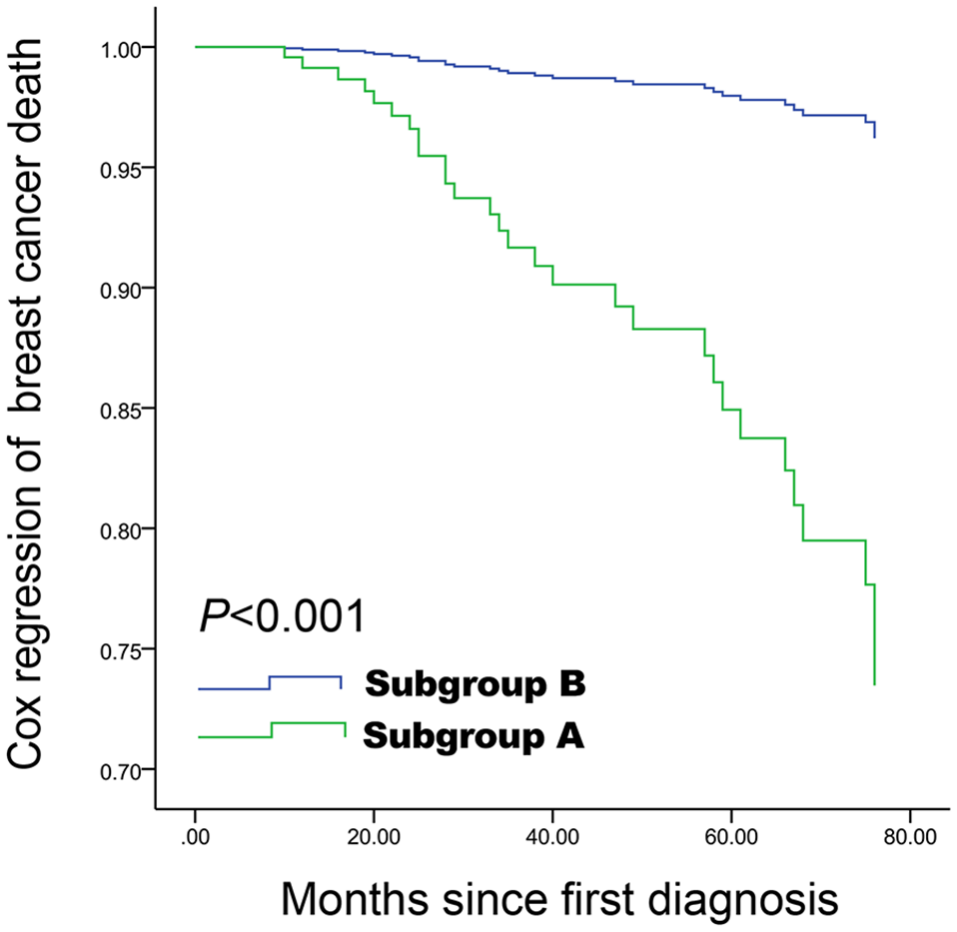
Cox proportional hazards curve for death in women with spiculated morphology and anti-parallel orientation compared with the remaining women, according to the other five covariables factors. Spiculated morphology and anti-parallel orientation ultrasound features are associated with poor breast cancer-specific survival, and a difference was still observed after adjustment for other covariables. Subgroup A: Lesions with both spiculated morphology and anti-parallel orientation; Subgroup B: All other patients.

Spiculated morphology and anti-parallel orientation (HR=7.45, 95%CI: 3.26-17.00, P<0.001), age ≥55 years (HR=5.94, 95%CI: 2.24-15.72, P<0.001), and lymph nodes metastasis (HR=3.99, 95%CI: 1.89-8.43, P<0.001) were independently associated with poor BCSS (**Table 3** and **Figure 3**). Spiculated morphologyand anti-parallel orientation (HR=6.42, 95%CI: 3.19-12.93, P<0.001), age ≥55 years (HR=1.98, 95%CI: 1.11-3.54, P=0.021), and lymph nodes metastasis (HR=2.99, 95%CI: 1.71-5.23, P<0.001) were independently associated with poor DFS (**Table 4**).

**Table 3:** Cox proportional hazards models of the factors for breast cancer death.

Variables		HR	95% CI	P
Age	≥55 vs. <55	5.94	2.24-15.72	<0.001
Lymph nodes metastasis	Positive vs. negative	3.99	1.89-8.43	<0.001
Sonography features	Subgroup A vs. B	7.45	3.26-17.00	<0.001

Subgroup A, lesions with both spiculated morphology and anti-parallel orientation; Subgroup B, all other patients.

HR, hazard ratio; CI, confidence interval.

**Table 4:** Cox proportional hazards models of the factors for disease-free survival.

Variables		HR	95% CI	P
Age	≥55 vs. <55	1.98	1.11-3.54	0.021
Lymph nodes metastasis	Positive vs. negative	2.99	1.71-5.23	<0.001
Sonography features	Subgroup A vs. B	6.42	3.19-12.93	<0.001

Subgroup A, lesions with both spiculated morphology and anti-parallel orientation; Subgroup B, all other patients.

HR, hazard ratio; CI, confidence interval.

When the risk factors were analyzed for tumors of 1-10 mm (n=158), the combination of spiculated morphology and anti-parallel orientation was the only factor independently associated with BCSS (HR=12.69; 95%CI: 2.83-56.84, P=0.001). Among the 158 women with breast cancers of 1-10 mm, six had spiculated morphology combined with anti-parallel orientation and accounted for 43% (3/7) of the breast cancer deaths. Positive lymph node metastasis (n=184 patients) was also associated with BCSS when tumors >10 mm (HR=3.99; 95% CI, 1.89-8.43, P<0.001).

## Discussion

This study aimed to explore whether ultrasound features could identify small breast cancers with poor outcomes. The results suggested that spiculated morphology and anti-parallel orientation at ultrasound were associated with poor BCSS and DFS in patients with breast cancer <20 mm.

The BCSS in the present study was 96.2%, which is consistent with previous studies in similar study populations (3–5). In the present study, lymph node metastasis was a statistically significant predictor for small cancer. While no significant differences were observed regarding tumor grade. Among the 30 breast cancer deaths, only 10 patients were diagnosed as grade 3 tumors. Grade 3 tumors only accounted for 24.8% (196/790) among the small breast cancers including in this study, which is consistent with the theory that pathological grade tend to progress towards a higher grade during breast cancer growth (22). This biological characteristic might explain the different value of tumor grade in predicting outcome among breast cancers with different sizes. It was reported that the involvement of lymph nodes is strongly related to the metastatic potential of breast cancer (22), and lymph node involvement in early stage, small breast cancers might reflect a more aggressive biological behavior (5, 23). In this study, positive lymph node metastases were associated with poor BCSS in tumors of 10-20 mm, but not in tumors of 1-10 mm, as few positive lymph nodes were detected among tumors of 1-10 mm (n=20), which is supported by Tabar et al. (13). Hence, the traditional prognostic factors might not be reliable in breast cancers <20 mm.

Ultrasound imaging, which reflects the underlying pathological and biological characteristics of the breast tumors, might be a complementary and reliable way to characterize small breast cancers. Previous studies (13, 14) identified the role of mammography features in identifying high-risk small breast cancers. The association between ultrasound features and triple-negative breast cancer death was examined, but only 50.6% were tumor <20 mm (24). Chung et al. (25) found no association between ultrasound features and outcome by dividing tumors into mass or non-mass without detailed sonography features. Still, research concerning the predictive value of sonographic findings for long-term outcomes for small tumors is insufficient. In this study, spiculated morphologyand anti-parallel orientation, alone or in combination, were related to cancer death and recurrence, and the differences remained significant after adjusting for age, tumor grade, lymph node status, adjuvant therapy, and molecular biomarker. Survival to breast cancer <20 mm is favorable except for tumors with spiculated morphologycombined with a anti-parallel orientation. It is proposed that a non-proportional growth is a suspicious indicator for malignancy and poor prognosis (19, 24, 26), which supports the results of the present study. Compared with ductal carcinoma in situ, invasive ductal carcinoma is more likely to have a non-proportional growth orientation (27). Guo et al. (19) assessed the value of ultrasound features in 336 breast cancers and supposed that vertical growth tends to correlate with high-risk breast cancer. At the pathological examination, indolent cancers generally show a homogeneous expansive growth, while a non-proportional growth at ultrasound reflects a heterogeneous infiltrating growth of malignant cells through lobule of mammary gland boundaries (24, 28, 29) and indicates a more aggressive biological behavior, particularly when it occurs early in breast cancer development.

In this study, the presence of spiculated morphology was another factor associated with poor prognosis. Results about the correlation between spiculated morphology and breast cancer prognosis remain conflicting. Wu et al. (30) proposed that spiculated margins are significantly related to positive HER2, a factor for high tumor grade and poor prognosis (31). While some studies consider spiculated morphology are more frequently associated with the low-grade tumor and positive ER and PR (21, 32). The conflicting results might be contributed to the analysis of breast cancers of different sizes. Besides, the long-term outcomes of small breast cancer were observed, and the association of spiculated morphology and cancer-related mortality was identified in this study, while most previous studies compared the imaging findings between different tumor attributes, including pathology characteristics and molecular biomarkers, which might explain the inconsistence. In terms of pathological manifestations, a spiculated margin is a reflection of the invasive growth of malignant cells into the normal surrounding tissues, indicating a high invasive capability (30, 33, 34). In addition, various growth factors (including the vascular endothelial growth factor and transforming growth factor) can be secreted by malignant cells to stimulate angiogenesis, infiltration, and metastasis (34, 35). An infiltrating growth early in tumor development might represent a more aggressive biological behavior and a graver prognosis, which was consistent to our results.

Casting-type calcification in mammography is a reliable predictor for a worse prognosis for small breast cancer (14), but no significant associations with BCSS and DFS were observed in the present study. It might be because of the sensitivity of ultrasound for calcifications. Molecular biomarkers are widely used in the clinical decision-making of breast cancer management, but no significant associations were observed between the biomarkers (ER, PR, HER2, and Ki67) and breast cancer outcomes in this study. Tryfonidis et al. (36) reported that small triple-negative and HER2-positive breast cancers tend to have a worse prognosis, while Galimberti et al. (37) and Bao et al. (7) proposed that there are no correlations between the status of ER, PR, HER2, and the outcome events. We are cautious about the results of the role of the molecular biomarker in breast cancer prognosis because of different cut-off values and a median follow-up time of only 3.5 years in this study, which might not be enough to observe the effects of molecular biomarkers on the outcomes of small breast cancer.

The treatment of each breast cancer is based on the careful consideration of recognized prognostic factors like tumor size, tumor grade, lymph node status, and molecular subtype. However, this decision-making assessment for small breast cancers might not be the same as for larger ones. Among the 30 breast cancer deaths in this study, 13 had negative lymph nodes, and 19 had grade 1-2 tumors, and one patient was even diagnosed with ductal carcinoma *in situ* at the final pathological examination. The combination of spiculated morphology and anti-parallel orientation was an independent predictor for breast cancer death in this study. Combining the imaging findings with the existing prognosis factors could contribute to the clinical decision-making by identifying the small breast cancers with a poor prognosis, for which more aggressive treatments might be warranted. Ultrasound is a readily available and inexpensive imaging modality that can be used alone or in combination with mammography. In a developing country where healthcare resources can be scarce in specific regions, standalone ultrasound can be used for breast cancer screening since some ultrasound systems are portable and mammography and magnetic resonance imaging might be unavailable.

There are some limitations to this study. First, it was a retrospective and single-center study, and bias might exist. Second, the classification of different ultrasound features is subjective and operator-dependent. Experienced radiologists are required to obtain reliable results. A high image resolution is required to observe the spiculated in small breast cancer. Third, the follow-up time was not long enough, and the number of breast cancer-specific death was small. Finally, as the number of deaths from cancer in the series is very low, there are very few findings that could be significant. Further studies are required.

## Conclusion

The combined presence of spiculated morphology and anti-parallel orientation in breast ultrasound might be associated with poor BCSS and DFS in patients with primary breast cancers <20 mm. Combining these imaging findings as complementary risk indicators with existing factors might improve the clinical evaluation in small breast cancers.

## Data availability statement

The original contributions presented in the study are included in the article. Further inquiries can be directed to the corresponding author.

## Ethics statement

This retrospective study was approved by the institutional ethics committee of Shanghai General Hospital. Written informed consent was waived by the institutional review board.

## Author contributions

Conceptualization: RW; Data curation: SHS; Formal analysis: SHS; Funding acquisition: RW; Investigation: CXL; Methodology: YZ; Project administration: MHY; Resources: XL; Software: JC; Supervision: RW; Validation: JFW; Roles/writing - original draft: SHS; Writing - review and editing: MHY.
